# Abnormal Osmotic Avoidance Behavior in *C*. *elegans* Is Associated with Increased Hypertonic Stress Resistance and Improved Proteostasis

**DOI:** 10.1371/journal.pone.0154156

**Published:** 2016-04-25

**Authors:** Elaine C. Lee, Heejung Kim, Jennifer Ditano, Dacie Manion, Benjamin L. King, Kevin Strange

**Affiliations:** 1 MDI Biological Laboratory, Salisbury Cove, ME, 04672, United States of America; 2 University of Connecticut, Storrs, CT, 06269, United States of America; Brown University/Harvard, UNITED STATES

## Abstract

Protein function is controlled by the cellular proteostasis network. Proteostasis is energetically costly and those costs must be balanced with the energy needs of other physiological functions. Hypertonic stress causes widespread protein damage in *C*. *elegans*. Suppression and management of protein damage is essential for optimal survival under hypertonic conditions. ASH chemosensory neurons allow *C*. *elegans* to detect and avoid strongly hypertonic environments. We demonstrate that mutations in *osm-9* and *osm-1*2 that disrupt ASH mediated hypertonic avoidance behavior or genetic ablation of ASH neurons are associated with enhanced survival during hypertonic stress. Improved survival is not due to altered systemic volume homeostasis or organic osmolyte accumulation. Instead, we find that *osm-9(ok1677)* mutant and *osm-9(RNAi)* worms exhibit reductions in hypertonicity induced protein damage in non-neuronal cells suggesting that enhanced proteostasis capacity may account for improved hypertonic stress resistance in worms with defects in osmotic avoidance behavior. RNA-seq analysis revealed that genes that play roles in managing protein damage are upregulated in *osm-9(ok1677)* worms. Our findings are consistent with a growing body of work demonstrating that intercellular communication between neuronal and non-neuronal cells plays a critical role in integrating cellular stress resistance with other organismal physiological demands and associated energy costs.

## Introduction

Cellular life requires precise control of protein structure and function, which are determined by protein conformation, concentration, assembly and localization. The homeostatic mechanisms that maintain protein function are collectively termed proteostasis. The proteostasis network is evolutionarily conserved and comprises the tightly integrated and regulated activities of gene transcription, RNA metabolism and protein synthesis, folding, assembly, trafficking, disassembly, repair and degradation [1–3].

Proteostasis is under constant challenge. Protein structure is inherently unstable [4,5] and readily disrupted by gene mutations and numerous environmental stressors. Random errors in cellular processes such as DNA replication, transcription and protein translation also disrupt protein structure. A poorly understood decline in the capacity of cellular proteostasis networks that repair and degrade damaged proteins is thought to underlie pathophysiology associated with senescence [6,7].

Proteostasis is energetically costly and those costs must be balanced with the energy needs of other cellular and organismal functions as well as with the requirement of the organism to respond to environmental change. A fundamental and emerging question is how do organisms ensure optimal survival by partitioning a finite energy budget to meet the demands of proteostasis and other essential physiological processes including reproduction? Several recent studies in *C*. *elegans* have pointed to a critical role for the nervous system and communication between different tissue types in coordinating organismal proteostasis needs. For example, Morimoto and co-workers have demonstrated that the heat shock response of non-neuronal cells is modulated by inhibitory inputs from AFD thermosensory neurons as well as neural circuits that respond to metabolic status and nutrient availability [8,9]. Activation of the endoplasmic reticulum (ER) unfolded protein response (UPR) in neurons activates the UPR in the intestine via a neurosecretory process that in turn increases organismal stress resistance and longevity [10]. Inhibition of protein translation or degradation in non-neuronal cells alters *C*. *elegans* behavioral responses that are controlled by chemosensory neurons [11].

The proteostasis network plays a critical role in ensuring optimal survival of *C*. *elegans* under dehydrating conditions. When exposed to hypertonic stress, *C*. *elegans* rapidly loses water and becomes paralyzed. Water loss is followed by systemic volume recovery and accumulation of the organic osmolyte glycerol [12]. Dehydration causes rapid and widespread protein aggregation and misfolding [13,14]. Stress induced protein damage is minimized by genes that function in protein degradation [13] and by reductions in protein synthesis [15,16]. Hypertonicity induced inhibition of translation also serves as a signal that activates glycerol accumulation pathways [17,18] and possibly other mechanisms that confer increased hypertonic stress resistance.

Given the importance of the proteostasis network to the survival of *C*. *elegans* in hypertonic environments, we characterized hypertonic stress resistance in worm strains with defects in osmotic avoidance behavior. *C*. *elegans* avoids strongly hypertonic solutions. This avoidance behavior is mediated by ASH chemosensory neurons [19,20]. We demonstrate that disruption of osmotic avoidance behavior via gene mutations or genetic ablation of ASH neurons is associated with enhanced survival in hypertonic environments. Enhanced survival is not due to altered systemic volume regulation or glycerol accumulation and instead may be due to enhanced proteostasis capacity.

## Materials and Methods

### *C*. *elegans* strains

The following strains were obtained from the *Caenorhabditis* Genetics Center (University of Minnesota, Minneapolis, MN, USA): wild-type N2 Bristol, VC1262 *osm-9(ok1677)*, MT3645 *osm-12(n1606)*, AM140 *rmIs132*[*Punc*-*54*::*Q35*::*YFP*], SD551[*let-60(ga89)*], NL790 *gpa-4(pk381)*, PY7505 *oyIs84*[*gpa-4p*::*TU#813 + gcy-27p*::*TU#814 + gcy-27p*::*GFP + unc-122p*::*dsRed*], TU3311 *uIs60*[*Punc-119*::*YFP* + *Punc-119*::*sid-1*], and *Is[sra-6p*::*mCasp]*. VC1262 and AM140 strains and TU3311 and SD551 strains were crossed to generate *osm-9(ok1677); rmIs132*[*Punc*-*54*::*Q35*::*YFP*] and *let-60(ga89);uIs60*[*Punc-119*::*YFP* + *Punc-119*::*sid-1*] worms, respectively. F2 progeny were selected for YFP expression and the presence of the *osm-9(ok1677)* or *let-60(ga89)* alleles were verified by PCR. *Is[sra-6p*::*mCasp]* [21] worms were a generous gift of Dr. Kazushi Yoshida. Standard osmotic avoidance assays [22] were carried out blinded to verify strains predicted or shown previously to be defective in osmotic avoidance behavior. Unless stated otherwise, worms were cultured at 20°C on nematode growth media (NGM) plates using standard methods [23]. Hypertonic agar plates were generated by adding NaCl to standard nematode growth medium.

### Fluorescent protein aggregate measurement

The number of body wall muscle cell Q35::YFP aggregates were quantified manually in blinded experiments using a Zeiss Stemi SV11 microscope (Chester, VA). Single aggregate volume measurements and fluorescence recovery after photobleaching (FRAP) analysis were carried using confocal microscopy as described previously [14].

*RNA interference*. RNA interference (RNAi) was performed by feeding worms from the L1 larval stage with bacteria expressing a nonspecific scrambled dsRNA or dsRNA specific to *osm-9*. Late stage L4 RNAi worms were transferred to control or high NaCl growth plates seeded with dsRNA expressing bacteria.

### *let-60(ga89)* mutant phenotype assay

Temperature sensitive *let-60(ga89)* mutant worms were maintained at the permissive temperature of 16°C. Defective egg hatching or larval arrest phenotypes were quantified by transferring 1-day old gravid adults to 300 mM NaCl feeding plates and then removing them after 24 h. Eggs were scored for failure to hatch or develop past the L1 larval stage.

### Survival assays

Synchronized late L4 worms were exposed to various stressors and survival was determined by prodding animals with a platinum wire. Worms were considered to be dead if they did not respond to repeated prodding. All survival studies were done blinded.

### Lifespan analysis

Synchronized L4 larvae were transferred to 51mM NaCl agar plates containing 50 μg/ml 5-fluorodeoxyuracil (FUDR; Sigma-Aldrich, St. Louis, MO). Animals were transferred onto fresh plates containing FUDR every 2 days for the first week, then every 4–5 days thereafter. Survival was scored daily for touch provoked movement.

### ^35^S-methionine labeling of total protein

Incorporation of ^35^S-methionine into total protein was used to assess rates of protein synthesis and degradation. Radiolabeling was carried out using methods similar to those described by others [24]. Briefly, synchronized L4 worms were fed ^35^S-methionine labeled OP50 bacteria for 4 h, washed and incubated with unlabeled OP50 for 1 h to purge radioactive intestinal bacteria, and then washed thoroughly with NGM buffer. Washed worms were flash frozen in liquid nitrogen and stored at -80°C before extraction. Protein was extracted from thawed samples by trichloroacetic acid-ethanol protein precipitation. Total protein concentration and radioactivity incorporation were measured by BCA assay (Pierce Biotechnology) and liquid scintillation counting, respectively.

### RNA-sequencing and gene expression analysis

N2 and *osm-9(ok1677)* worms were carefully staged for gene expression analysis. Staging was carried out using well established methods [25] and took into account the slight developmental delay we observed in *osm-9(ok1677)* worms. Briefly, bleached eggs were placed on clean NGM plates to synchronize L1 stage larvae. After washing, L1 larvae were transferred to NGM plates seeded with OP50 and grown at 20°C. Cultures were visually inspected frequently during the staging process. Late stage L4 larvae were defined by the appearance of a white crescent shape that surrounds the prospective vulva. To avoid gene expression changes that might be associated with the L4/adult molt, animals with white crescents were transferred to experimental conditions while they were still moving and feeding and had not yet entered the quiescent period that occurs prior to molting [26].

L4 stage N2 and *osm-9(ok1677)* worms were washed and transferred to control (51mM NaCl) and hypertonic stress (200mM NaCl) agar plates for 6 h. Two to four independent experiments were conducted for each condition. After the exposure period, plates were visually inspected. No evidence of molting or development of worms into gravid adults was detected. Worms were rinsed off plates, washed 4 times, pelleted, and snap frozen for storage at -80°C until processing. Total RNA was isolated using TRIzol (Invitrogen, Carlsbad, CA). RNA quality was assessed by spectrophotometry and by using an RNA 6000 Nano Kit and Bioanalyzer 2100 (Agilent, Santa Clara, CA).

RNA-sequencing was carried out by Ocean Ridge Biosciences (Palm Beach Gardens, FL). llumina paired-end and barcoded TrueSeq mRNA sequencing libraries were prepared from RNA samples with an RNA Integrity Numer (RIN) of ≥8.0, pooled and sequenced for 76 cycles on two Illumina HiSeq2500 flow cell lanes (Illumina, Inc., San Diego, CA). Following diagnostic analyses of the 532,462,712 sequence reads using FastQC (http://www.bioinformatics.babraham.ac.uk/projects/fastqc/), sequences were aligned to the WS235 version of the *C*. *elegans* genome assembly using Tophat version 1.4.1 [27] and mapped to Ensembl (version 71) [28] annotated genes using Cufflinks version 2.0.1 [29]. Differentially expressed genes were determined using read counts per gene and a P-value threshold of ≤0.05 with edgeR version 3.2.4 [30] for each pairwise comparison among sample groups. Functional annotation for genes was obtained using WormBase [31] and Ensembl BioMart [32]. Enrichment analysis of DAF-16 and SKN-1 identified by Tepper et al. (33) and Niu et al. [34] was conducted using Fisher’s Exact Test in R 3.2.0. RNA-Seq data are available at the NCBI Gene Expression Omnibus (http://www. ncbi.nlm.nih.gov/geo/) under accession GSE73589 and at the NCBI Short Read Archive (http://www.ncbi.nlm.nih.gov/sra) under accession SRP064324.

### Statistical analysis

All data are presented as means ± S.E. Statistical significance was determined using Student’s two tailed t-test when two means were compared or ANOVA with Tukey post hoc test when comparing multiple means. P values of ≤0.05 were taken to indicate statistical significance.

## Results

Mutant worms with defective osmotic avoidance behavior are termed Osm for “osmotic avoidance abnormal”. Ten *osm* genes have been identified. Loss of *osm-7*, *osm-8* and *osm-11* function to activate *gpdh-1* expression and induce constitutive glycerol accumulation [17,35–37]. All other *osm* genes are required for the normal function or development of chemosensory neurons. *osm-9* and *osm-12* encode a TRPV cation channel [20] and a protein required for the biogenesis of sensory neuron cilia [38], respectively. Both genes are expressed in ASH osmosensory neurons as well as other chemosensory neurons [39].

**Fig 1:**
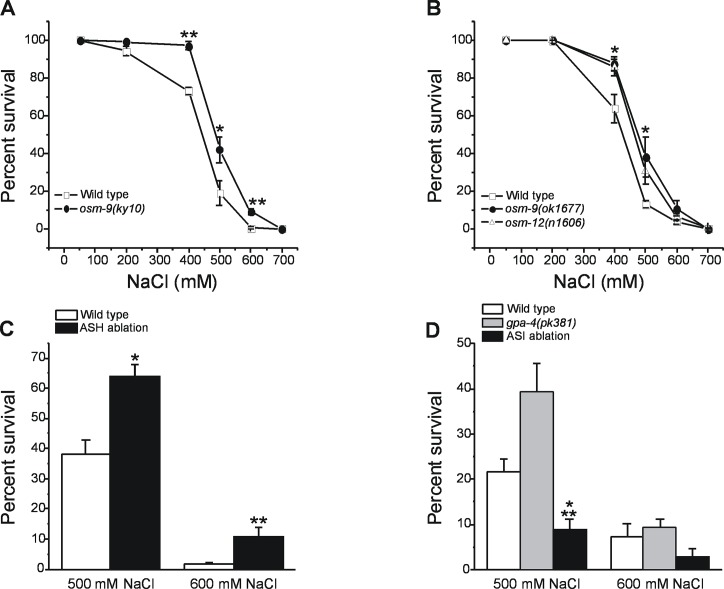
Effect of *osm-9* and *osm-12* mutations and genetic ablation of ASH osmosensory neurons on survival during hypertonic stress. (A) Survival of wild type worms and *osm-9(ky10)* loss-of-function mutants exposed to different concentrations of NaCl for 24 h. Values are means ± S.E. of four independent and blinded experiments. *P<0.05 and **P<0.001 compared to wild type animals. (B) Survival of wild type worms and *osm-9(ok1677)* and *osm-12(n1606)* loss-of-function mutants exposed to different concentrations of NaCl for 24 h. Values are means ± S.E. of four independent experiments. *P<0.05 for both *osm-9(ok1677)* and *osm-12(n1606)* mutants compared to wild type animals. (C) Survival of wild type and ASH ablated (i.e., *Is[sra-6p*::*mCasp1]* transgenic strain) worms exposed to 500 or 600 mM NaCl for 24 h. Values are means ± S.E. of 6–12 independent experiments. *P<0.002 and **P<0.02 compared to wild type worms. (D) Survival of wild type, *gpa-4(pk381)* mutants and ASI ablated (i.e., *oyIs84*[*gpa-4p*::*TU#813 + gcy-27p*::*TU#814 + gcy-27p*::*GFP + unc-122p*::*dsRed*] transgenic strain) worms exposed to 500 or 600 mM NaCl for 24 h. Values are means ± S.E. of 8–9 experiments. *<P<0.05 compared to wild type and **P<0.001 compared to ASI ablation.

To determine whether defects in osmotic avoidance behavior impact hypertonic stress resistance, we initially examined the ability of wild type N2 and the *osm-9(ky10)* mutant to survive a 24 h exposure to high NaCl growth plates. The *ky10* allele is a widely studied loss-of-function mutation that disrupts a variety of chemosensory behaviors including avoidance of hypertonic environments [20]. As shown in Fig 1A, *osm-9(ky10)* mutants had significantly (P<0.05) greater survival at 400, 500 and 600 mM NaCl compared to wild type animals.

We also examined hypertonic stress resistance of *osm-9(ok1677)* and *osm-12(n1606*) mutants. *osm-9(ok1677)* is a deletion mutant that can be followed in crosses using PCR. *osm-9(ok1677)* worms developed and moved somewhat more slowly than *osm-9(ky10)* worms. However, all three genotypes exhibited similar defects in osmotic avoidance behavior (Table 1). Compared to wild type worms, both *osm-9(ok1677)* and *osm-12(n1606*) worms showed significantly (P<0.05) higher survival at 400 and 500 mM NaCl (Fig 1B).

**Table 1:** Osmotic avoidance behavior in wild type and ASH ablation worms and *osm* mutants.

Genotype	Fraction with defective osmotic avoidance
Wild type	0.09 ± 0.03 (13)*
*osm-9(ky10)*	0.80 ± 0.10 (7)
*osm-9(ok1677)*	0.67 ± 0.07 (7)
*Is[sra-6p*::*mCasp]*	0.78 ± 0.03 (6)
*osm-12(n1606)*	0.60 ± 0.05 (4)

Values are means ± S.E. (n).

*P<0.001 compared to *osm* mutants and ASH ablation strain. Osmotic avoidance was not significantly (P>0.05) different in *osm* mutants and ASH ablation worms.

Acclimation of *C*. *elegans* to relatively low levels of hypertonic stress increases survival under more extreme conditions. Increased survival is due to reduced water loss that results from organic solute accumulation and to enhanced proteostasis capacity [12–15]. We acclimated wild type, *osm-9(ok1677)* and *osm-12(n1606*) worms beginning at the L1 larval stage to 200 mM NaCl and then assessed survival in late L4 and young adult animals exposed to increasing NaCl levels. As expected, acclimation improved hypertonic stress resistance, but enhanced resistance was similar in wild type and mutant worms (data not shown).

Yoshida et al. [21] recently described a transgenic worm strain in which ASH neurons are ablated by expression of mouse caspase 1 driven by the promoter *sra-6*. *sra-6* is expressed highly in ASH neurons and weakly in ASI sensory and PVQ interneurons [39]. The *Is[sra-6p*::*mCasp]* transgenic has defects in avoidance of noxious chemicals [21] and also exhibits defects in avoidance of hypertonic environments similar to *osm-9(ky10)*, *osm-9(ok1677)* and *osm-12(n1606)* worms (Table 1).

We tested the effect of hypertonic stress on survival of wild type worms and the *Is[sra-6p*::*mCasp]* transgenic strain. The transgenic strain showed significantly (P<0.02) increased survival when exposed to 500 or 600 mM NaCl for 24 h (Fig 1C).

*osm-9*, *osm-12* and *sra-6* are expressed in ASH as well as other neuron types [39]. While not definitive, the only cell types in which expression of these three genes has been shown to overlap are ASH and ASI chemosensory neurons [39]. It is thus conceivable that the enhanced survival of *osm* mutants and the ASH ablation strain is due to defects in ASH and/or ASI neuron function. To assess the role of ASI neurons in hypertonic stress resistance, we quantified survival of *gpa-4(pk381)* loss-of-function mutants and the PY7505 transgenic strain in 500 and 600 mM NaCl. *gpa-4* encodes a G-protein alpha subunit and has been reported to be expressed selectively in ASI neurons [40]. In the PY7505 strain, the ASI neurons are ablated by transgenic caspase expression [41]. At 500 mM NaCl, ASI ablation worms showed significantly (P<0.05) reduced survival compared to either wild type N2 worms or *gpa-4(pk381)* mutants (Fig 1D). No significant (P>0.2) differences in survival between strains were observed at 600 mM NaCl (Fig 1D). Taken together, data in Fig 1 suggest that loss of ASH neuron functions required for avoidance of hypertonic environments is associated with enhanced hypertonic stress resistance.

Cells and organisms lose water when exposed to hypertonic conditions. Survival in hypertonic environments is dependent on three processes: 1) recovery of cell and systemic fluid volume via uptake of inorganic ions and water, 2) replacement of accumulated inorganic ions with small, non-perturbing solutes termed organic osmolytes, and 3) repair and/or removal of cellular and molecular damage induced by hypertonic stress. The enhanced osmotolerance of ASH neuron mutants could be due to enhanced activity of any combination of these processes. We therefore assessed the role of each of each pathway in the improved osmotolerance of *osm-9(ok1677)* mutants.

**Fig 2:**
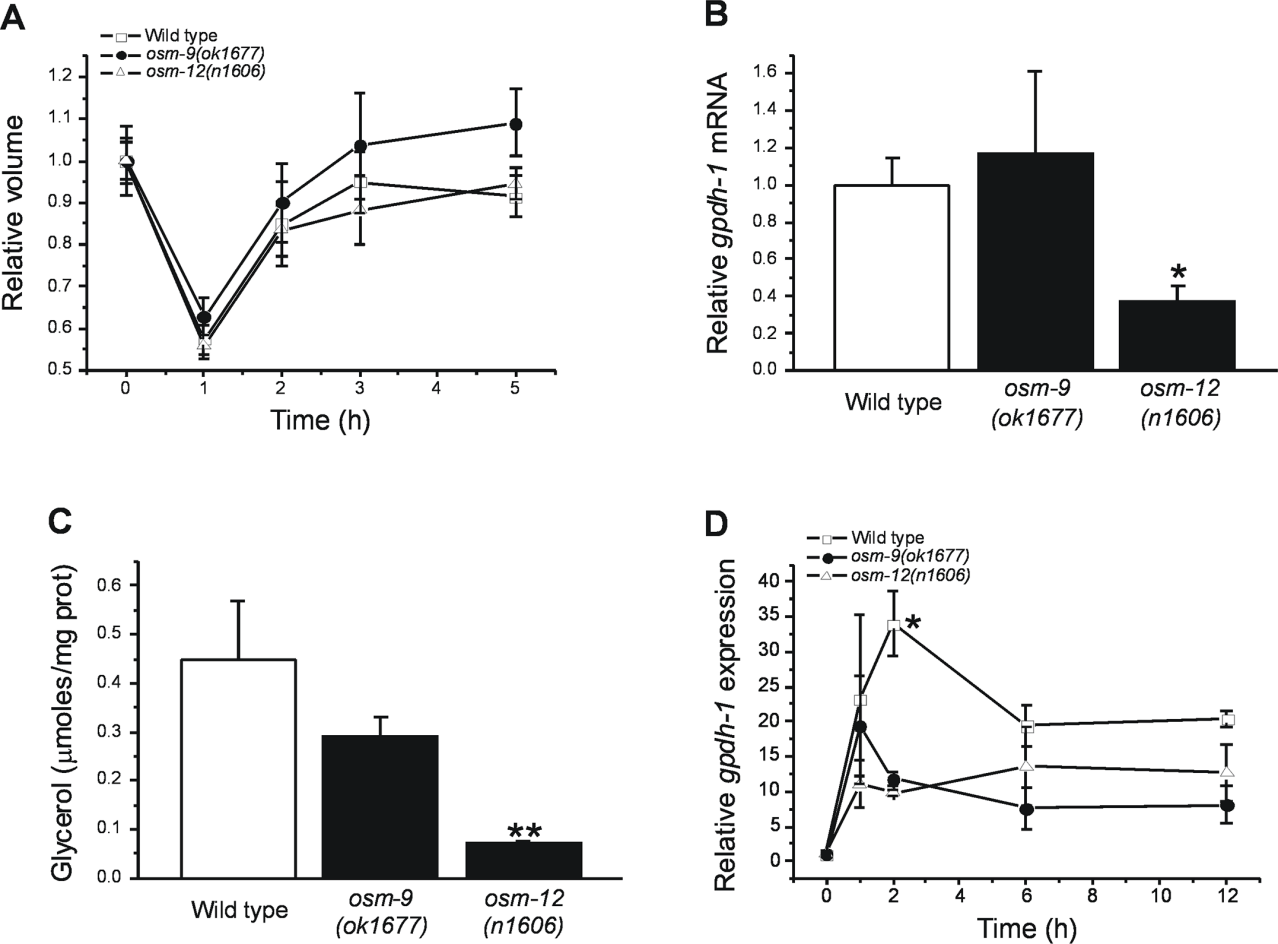
Effect of loss of ASH osmosensory neuron function on hypertonic stress induced whole animal water loss and volume recovery. (A) Whole worm volume changes. Wild type worms and *osm-9(ok1677)* and *osm-12(n1606)* loss-of-function mutants were transferred to agar plates containing 200 mM NaCl at time 0. Values are means ± S.E. (n = 8). *gpdh-1* mRNA expression (B) and whole animal glycerol levels (C) in wild type and *osm-9(ok1677)* and *osm-12(n1606)* worms. Values are means ± S.E. (n = 3). *P<0.002 and **P<0.007 compared to wild type worms. (D) Time course changes in *gpdh-1* expression. Wild type worms and *osm-9(ok1677)* and *osm-12(n1606)* loss-of-function mutants were transferred to agar plates containing 200 mM NaCl at time 0. Values are means ± S.E. (n = 3–5). *P<0.01 compared to *osm-9(ok1677)* and *osm-12(n1606)* mutants.

When exposed to hypertonic conditions, *C*. *elegans* rapidly loses water and becomes paralyzed. However, over a period of a few hours, systemic volume and normal motility is recovered. As shown in Fig 2A, N2 worms and *osm-9(ok1677)* and *osm-12(n1606)* mutants exhibit similar degrees of shrinkage and rates of volume recovery when exposed to agar plates containing 200 mM NaCl.

Increased transcription of *gpdh-1* catalyzes constitutive accumulation of the organic osmolyte glycerol in worms harboring loss-of-function mutations in certain *osm* genes [17,35–37]. Increased glycerol levels will reduce water loss, which in turn reduces cellular and molecular damage and increases survival under hypertonic conditions. To directly assess the role glycerol accumulation plays in the enhanced osmotolerance of *osm-9(ok1677)* and *osm-12(n1606)* mutants, we quantified *gpdh-1* expression and whole animal glycerol levels under control conditions (51mM NaCl). As shown in Fig 2B, *gpdh-1* mRNA levels were not significantly (P>0.7) different in N2 and *osm-9(ok1677)* mutant. In *osm-12(n1606)* mutants, *gpdh-1* mRNA levels were significantly (P<0.002) reduced compared to wild type worms. Glycerol levels (Fig 2C) mirrored *gpdh-1* expression and were not significantly (P>0.2) different in *osm-9(ok1677)* mutants. In *osm-12(n1606)* mutants, glycerol levels were reduced significantly (P<0.007) compared to wild type worms.

GPDH-1 catalyzes the rate limiting step in glycerol accumulation during hypertonic stress [17]. The similar rates of volume recovery between wild type, *osm-9(ok1677)* and *osm-12(n1606)* animals shown in Fig 2A indicate that enhanced survival of *osm* mutants is not due to increased *gpdh-1* expression and increased glycerol accumulation. To address this issue directly, we quantified temporal changes in *gpdh-1* expression during exposure of worms to 200 mM NaCl. *gpdh-1* expression increases rapidly during hypertonic stress and then declines to new steady state levels [18]. As shown in Fig 2D, *gpdh-1* expression showed similar rates of increase and steady state levels in wild type and *osm* mutants. However, the decline in *gpdh-1* expression observed 2 h after hypertonic stress was significantly (P<0.01) more rapid in *osm-9(ok1677)* and *osm-12(n1606)* animals. We conclude that enhanced volume and glycerol homeostasis do not contribute to increased short term hypertonic stress resistance in worms with defects in osmotic avoidance behavior.

In a recent genome-wide RNAi screen, we identified 40 genes whose function is essential for survival of *C*. *elegans* in hypertonic environments. Loss of these genes results in a **h**ypert**o**nic **s**ensitive or Hos phenoytype. Twenty *hos* genes play central roles in trafficking and destroying damaged proteins [13]. We subsequently demonstrated that hypertonic stress causes rapid, diverse and widespread protein damage [13–16]. Worms acclimated to mild hypertonic stress show enhanced survival and greatly reduced protein damage during exposure to more extreme hypertonicity [13–15]. Experimental maneuvers that increase protein damage reduce survival under hypertonic conditions [13,16]. Together, these studies indicate that detection and repair and/or destruction of damaged proteins is required for optimal survival during water loss, and suggest that enhanced proteostasis capacity may account for the increased osmotolerance of *osm-9(ok1677)* and *osm-12(n1606)* mutants.

**Fig 3:**
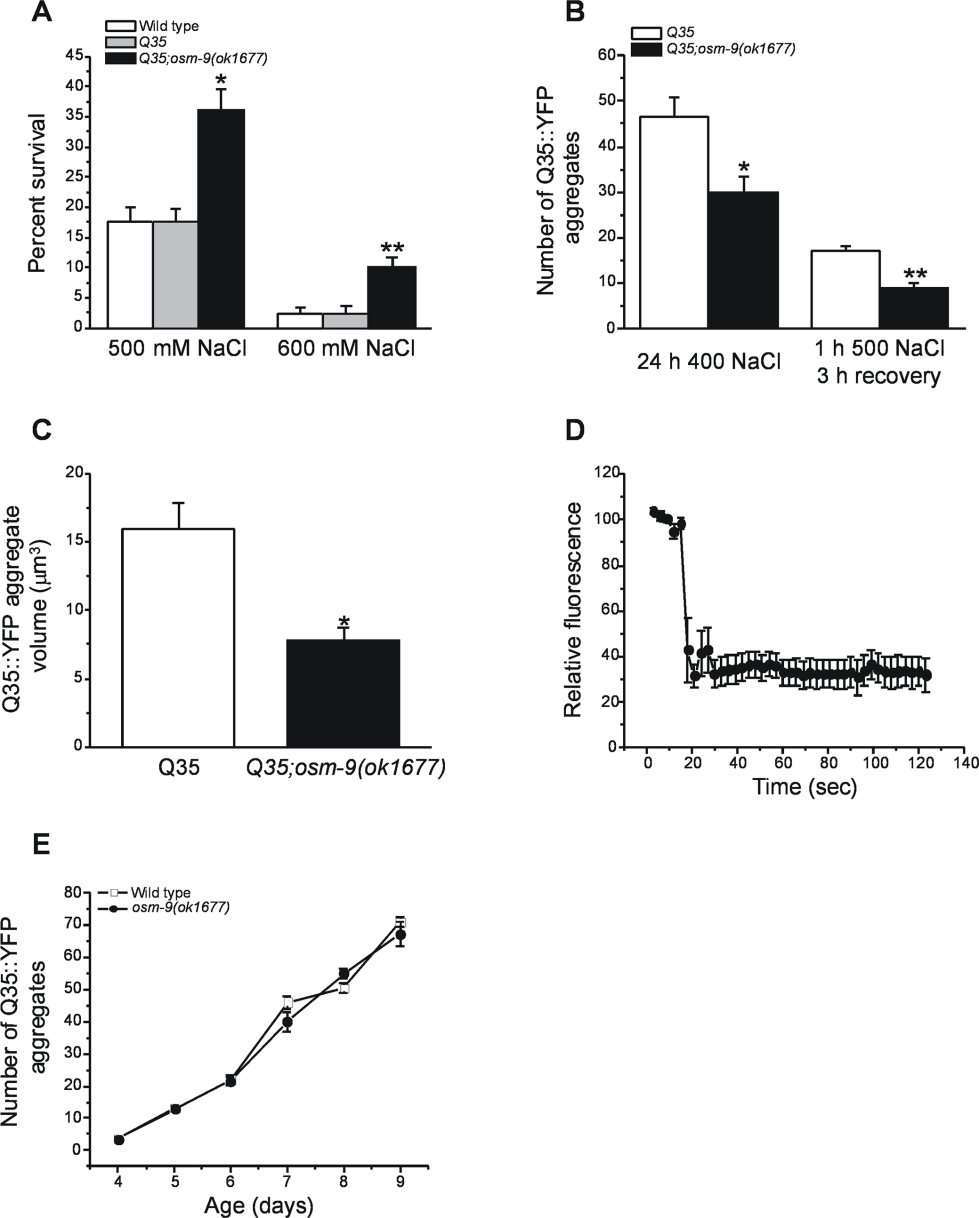
Hypertonic stress induced aggregation of Q35::YFP in body wall muscle cells of *osm-9(ok1677)* mutant worms. (A) Survival of wild type N2, Q35::YFP and Q35;*osm-9(ok1677)* worms exposed to 500 or 600 mM NaCl for 24 h. Values are means ± S.E. of 6 experiments. *P<0.001 and **P<0.01 compared to wild type N2 and Q35 worms. (B) Hypertonic stress induced Q35::YFP aggregation in wild type Q35 worms and *Q35;osm-9(ok1677)* mutants. Values are means ± S.E. of 4 experiments with a total of 32–78 worms. The number of Q35::YFP aggregates was quantified in worms exposed to 400 mM NaCl for 24 hour or 500 mM NaCl for 1 h followed by 3 h recovery on control medium. Values are means ± S.E. (n = 3). *P<0.005 and **P<0.001 compared to wild type Q35 worms. (C) Q35::YFP volume in wild type Q35 worms and *Q35;osm-9(ok1677)* mutants. Worms were exposed to 500 mM NaCl for 1 h and then allowed to recover on control medium before aggregate volumes were quantified. Values are means ± S.E. (n = 20 aggregates in 5–6 worms). *P<0.0004 compared to wild type Q35 worms. (D) Time course of bleaching and fluorescence recovery of Q35::YFP aggregates in *Q35;osm-9(ok1677)* mutant worms. Aggregates were induced by exposing worms to 500 mM NaCl for 1 h. FRAP analysis was performed 3 h after worms were returned to 51 mM NaCl medium. Values are means ± S.E. (n = 7 aggregates in 4 worms). (E) Time course of aging induced Q35::YFP aggregation in wild type Q35 worms and *Q35;osm-9(ok1677)* mutants. Values are means ± S.E. (n = 3).

To assess whether proteostasis capacity is altered in worm strains with defects in hypertonic avoidance behavior, we crossed *osm-9(ok1677)* mutant worms with a worm strain expressing polyglutamine (Q35) containing yellow fluorescent (YFP) protein in their body wall muscle cells. As shown in Fig 3A, wild type N2 and Q35::YFP worms showed similar survival when exposed to 500 or 600 mM NaCl. In contrast, survival was strongly enhanced by crossing *osm-9(ok1677)* into the Q35 strain, a finding consistent with results shown in Fig 1.

Q35::YFP is normally fully soluble in the muscles cells of young worms, but undergoes rapid aggregation when the animals are exposed to hypertonic media [13–15]. We quantified Q35::YFP aggregation in *osm-9(ok1677)* mutants exposed to 400 mM NaCl for 24 h or exposed to 500 mM NaCl for 1 h followed by a 3 h after recovery on control medium. As shown in Fig 3B, Q35::YFP aggregation was reduced 40–50% (P<0.005) in *osm-9(ok1677)* mutants. Total Q35:YFP fluorescence was not significantly (P>0.9) different in wild type versus *osm-9(ok1677)* mutants (total YFP fluorescence expressed as arbitrary units was 465 ± 41 and 469 ± 33 in wild type and *osm-9(ok1677)* worms, respectively; n = 11) demonstrating that differences in aggregation were not due to differences in Q35:YFP expression levels.

We also characterized the functional properties of the Q35::YFP aggregates. Aggregate volume increases rapidly when worms are exposed to hypertonic stress [14]. As shown in Fig 3C, aggregate growth was inhibited ~50% (P<0.0004) in *osm-9(ok1677)* mutants. However, the *osm-9(ok1677)* mutation had no effect on the solubility of aggregated Q35::YFP proteins. The fluorescence of regions within aggregates did not recover after photobleaching demonstrating that the aggregated proteins are insoluble (Fig 3D).

Finally, we quantified aging induced protein aggregation. Q35::YFP undergoes a slow, progressive aggregation as *C*. *elegans* ages [42]. As shown in Fig 3E, the *osm-9(ok1677)* mutation had no effect on the accumulation of Q35::YFP aggregates in aging worms. Taken together, data in Fig 3 demonstrate that hypertonicity induced Q35::YFP aggregation is reduced in *osm-9(ok1677)* mutant worms.

Temperature sensitive (ts) mutations give rise to proteins that fold and function correctly at low or ‘permissive’ temperatures [43–45]. *let-60(ga89)* encodes a ts mutant of ras GTPase. We have shown previously that hypertonic stress causes apparent misfolding of the *let-60(ga89)* encoded protein under permissive temperature conditions (16°C) and gives rise to an egg hatching and larval arrest phenotype [14,16].

**Fig 4:**
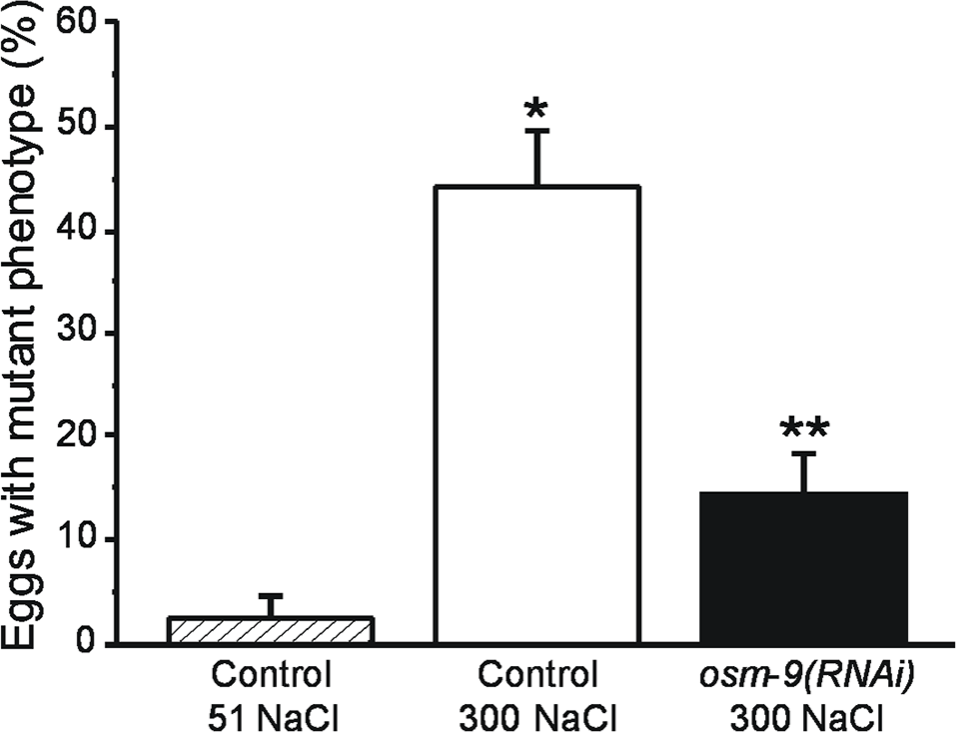
Effect of RNAi induced loss of *osm-9* function on *let-60(ga89)* induced egg hatching defects and larval arrest. *let-60(ga89)* encodes a ts mutant of ras GTPase. The mutant phenotype can be induced at permissive temperatures (16°C) by exposing worms to 300 mM NaCl [14,16]). *let-60(ga89)* mutants were crossed with *uIs60*[*Punc-119*::*yfp* + *Punc-119*::*sid-1*] worms to increase the sensitivity of neurons to RNAi (46). *let-60(ga89);uIs60*[*Punc-119*::*yfp* + *Punc-119*::*sid-1*] worms were fed bacteria expressing scrambled (control) or *osm-9* dsRNA and maintained at 16°C on agar plates containing either 51 or 300 mM NaCl. Values are means ± S.E. (n = 3–5 experiments with 100–300 eggs). *P<0.006 compared to control worms exposed to 51 mM NaCl. **P<0.005 compared to control worms exposed to 300 mM NaCl.

To determine whether disruption of genes that control hypertonic avoidance behavior modulates hypertonic stress induced protein misfolding, we examined the effect of loss of *osm-9* function on the *let-60(ga89)* mutant phenotype. *osm-9* and *let-60* are both located on chromosome IV and we were unable to successfully cross worm strains carrying mutant alleles of the two genes. We therefore utilized an RNA interference (RNAi) approach. *let-60(ga89)* mutants were crossed with the worm strain TU3311 *uIs60*[*Punc-119*::*yfp* + *Punc-119*::*sid-1*], which exhibits increased sensitivity of neurons to RNAi [46]. *let-60(ga89);uIs60*[*Punc-119*::*yfp* + *Punc-119*::*sid-1*] worms were fed bacteria expressing scrambled (control) or *osm-9* dsRNA. As expected, exposure of worms fed scrambled dsRNA at 16°C to 300 mM NaCl caused a striking and significant (P<0.006) increase in egg hatching defects and larval arrest. Silencing of *osm-9* function by RNAi reduced the expression of the mutant phenotype by ~70% (P<0.005) (Fig 4).

**Fig 5:**
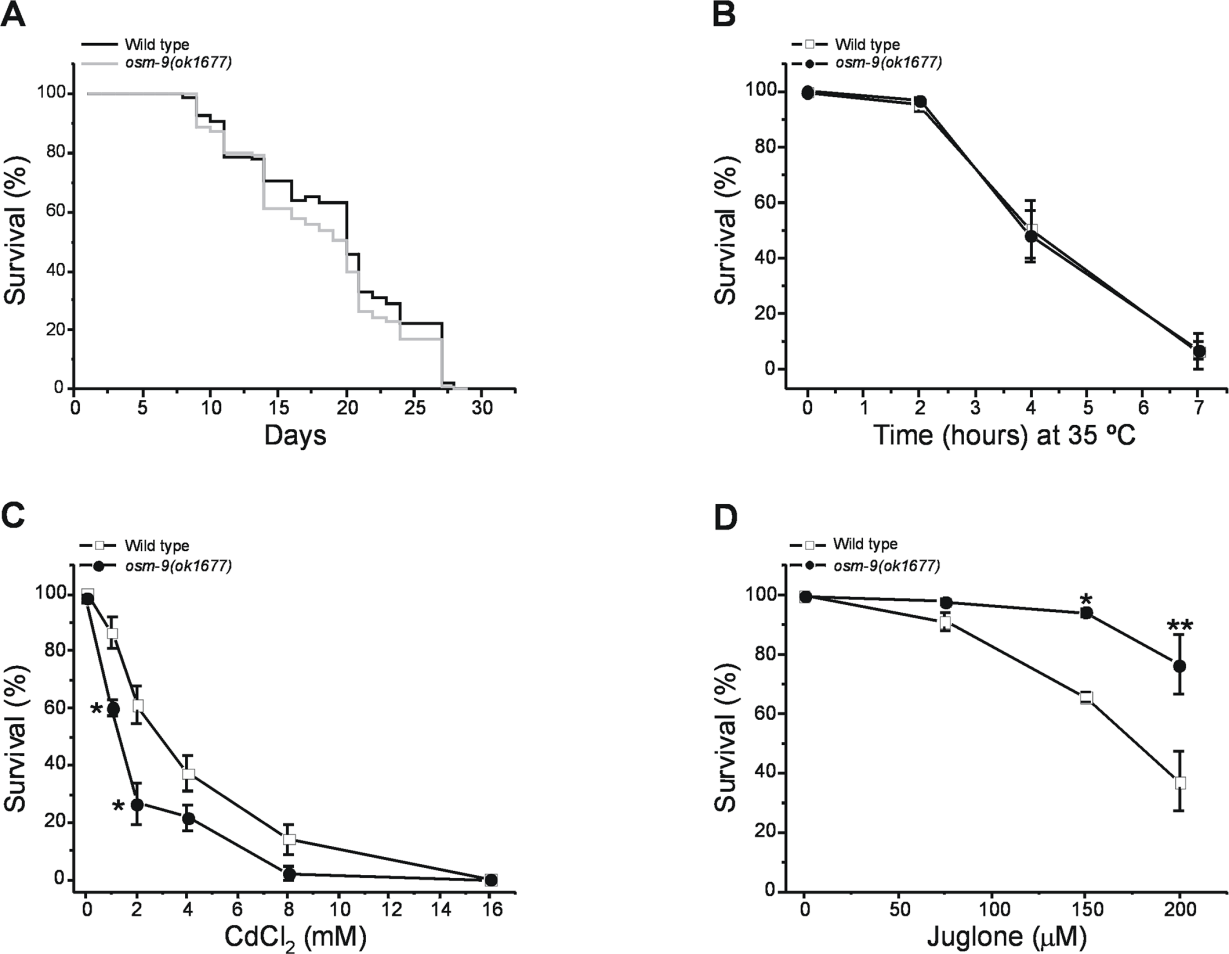
**Lifespan and (A) and resistance to heat shock (B), cadmium (C) and oxidative stress (D) in *osm-9(ok1677)* worms.** Median lifespan (A) was 21 days for wild type and *osm-9(ok1677)* worms (n = 100 worms for both groups). Values in B, C and D are means ± S.E. (n = 5). *P<0.007 and **P<0.04 compared to wild type worms.

Acclimation to hypertonic stress increases lifespan and resistance to other environmental stressors [47]. Given the improved hypertonic stress resistance and proteostasis capacity of *osm-9(ok1677)* mutant worms, we therefore also characterized the lifespan of this strain and its resistance to heat, heavy metal and oxidative stress. Median and maximum lifespan were not significantly (P>0.3) different in wild type and *osm-9(ok1677)* mutants (Fig 5A). The *osm-9(ok1677)* mutant exhibited sensitivity to heat shock similar to that of wild type animals (Fig 5B) and increased sensitivity to the heavy metal cadmium (Fig 5C). Resistance to high concentrations of the quinone juglone was significantly (P<0.04) increased in *osm-9(ok1677)* worms (Fig 5D). Quinones like juglone generate reactive oxygen species and form adducts with diverse macromolecules [48].

**Fig 6:**
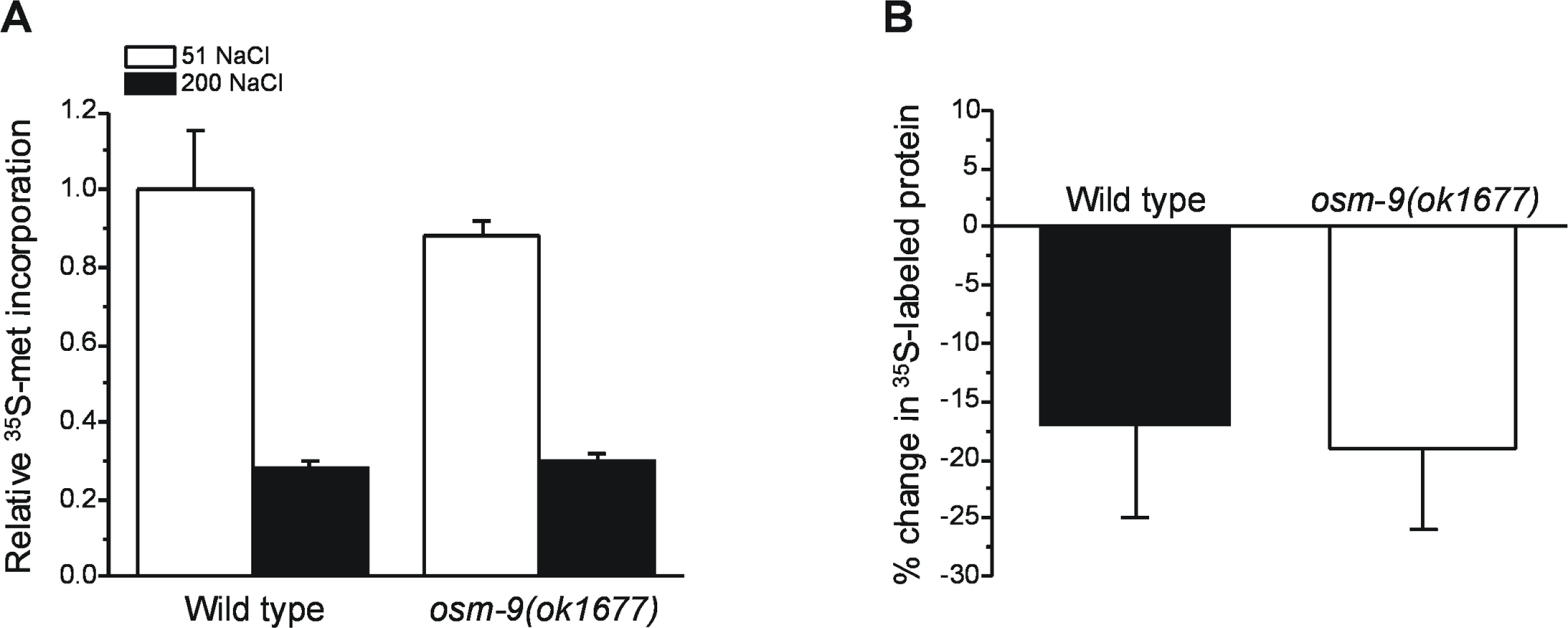
**Protein synthesis (A) and degradation (B) in *osm-9(ok1677)* worms.** Values are means ± S.D. (n = 2 independent experiments).

The enhanced proteostasis capacity of *osm-9(ok1677)* mutants could result from a number of different physiological changes. When exposed to hypertonic stress, protein synthesis is rapidly reduced in *C*. *elegans* and this reduction in translation functions in part to minimize protein damage [15,16,18]. More extensive reductions in translation could reduce protein damage in *osm-9(ok1677)* mutants. However, reductions in rates of translation were not significantly different (P>0.7) in wild type and *osm-9(ok1677)* worms exposed for 2 h to 200 mM NaCl (Fig 6A). Net rates of total protein degradation, measured as the decrease in ^35^S-methionine labeling following a 6 h exposure to 200 mM NaCl in the presence of 500 μg/ml cycloheximide, were also not significantly (P>0.7) different in the two groups of animals (Fig 6B).

The improved proteostasis capacity of the *osm-9(ok1677)* worm strain could be due to altered expression of genes encoding components of the proteostasis network. To begin testing this possibility, we performed RNA-sequencing gene expression analyses on wild type and *osm-9(ok1677)* worms under control and hypertonic stress conditions. In unstressed *osm-9(ok1677)* worms we found significant (P<0.05) upregulation of genes that have well defined or likely roles in protein degradation, synthesis and folding as well as genes with RNAi phenotypes of increased protein aggregation, altered sensitivity to protein aggregation and/or decreased hypertonic stress resistance. For example, in control *osm-9(ok1677)* worms we identified 23 upregulated (1.4- to 3.2-fold) genes that play known or presumptive roles in protein degradation including proteases, proteins associated with lysosome function, E3 ubiquitin ligases and E2 ubiquitin-conjugating enzymes (Table 2). RNAi silencing of one of these genes, *vha-3*, a vacuolar proton ATPase subunit likely required for lysosome function, results in increased protein aggregation during hypertonic stress and decreased hypertonic stress resistance [13]. Knockdown of *C17H11*.*6*, which encodes a predicted E3 ubiquitin ligase, increases sensitivity to protein aggregation-induced paralysis [49].

**Table 2:** Putative proteostasis genes differentially upregulated in *osm-9(ok1677)* worms under control conditions.

Sequence name	Gene	Brief description and RNAi phenotypes	*osm-9(ok1677)* vs. WT fold change	modENCODE ChIP-Seq Peaks
**Protein degradation**				
C05E4.1	*srp-2*	Serpin serine protease inhibitor	3.2	
C02B4.1	*adt-1*	Metalloproteinase	2.9	
R11A5.7	*suro-1*	Peptidase	2.8	DAF-16
F44B9.1	*dpf-6*	Peptidase	2.3	
C52E4.1	*cpr-1*	Cathepsin B-like cysteine protease	2.1	
F41E6.6	*tag-196*	Protease	2.0	DAF-16
Y43F4A.1	*Y43F4A*.*1*	Peptidase	1.9	
C04A11.4	*adm-2*	Metalloproteinase	1.9	SKN-1, DAF-16
ZK20.6	*nep-1*	Metalloproteinase	1.9	DAF-16
LLC1.1	*tra-3*	Calpain type protease; lysosome function	1.8	
T24A11.3	*toh-1*	Metalloproteinase	1.8	SKN-1, DAF-16
ZK970.1	*nep-26*	Metalloproteinase	1.8	
Y39A3CL.5	*clp-4*	Calpain type protease	1.7	DAF-16
F11A6.1	*kpc-1*	Protease	1.7	SKN-1, DAF-16
Y60A3A.1	*unc-51*	Serine/threonine kinase; autophagy	1.5	
K11D2.2	*asah-1*	Acid ceramidase; lysosome function	2.5	
T14F9.3	*hex-1*	Beta-hexosaminidase; lysosome function	1.5	
Y38F2AL.4	*vha-3*	Vacuolar proton ATPase subunit; lysosome function; increased protein aggregation and increased sensitivity to hypertonic stress [13]	1.5	
Y37E11AR.2	*siah-1*	E3 ubiquitin ligase	3.3	SKN-1
Y67D8C.5	*eel-1*	E3 ubiquitin ligase	2.2	DAF-16
C17H11.6	*C17H11*.*6*	E3 ubiquitin-protein ligase; hypersensitive to protein aggregation-induced paralysis [49]	1.6	
C35B1.1	*ubc-1*	E2 ubiquitin-conjugating enzyme	1.5	
Y71G12B.15	*ubc-3*	E2 ubiquitin-conjugating enzyme	1.4	SKN-1, DAF-16
**Protein synthesis**				
R10E4.2	*sup-26*	mRNA binding protein; regulation of translation	2.7	
Y57A10A.30	*ife-5*	Translation initiation	2.2	DAF-16
R03G5.1	*eef-1A*.*2*	Protein elongation; increased protein aggregation [50]	1.7	DAF-16
**Protein folding**				
C50F2.6	*fkb-5*	Peptidyl-prolyl cis-trans isomerase (FK506-binding protein family)	3.1	DAF-16
ZC455.10	*fkb-4*	Peptidyl-prolyl cis-trans isomerase (FK506-binding protein family)	3.0	
Y18D10A.25	*fkb-8*	Peptidyl-prolyl cis-trans isomerase (FK506-binding protein family)	2.9	
C05C8.3	*fkb-3*	Peptidyl-prolyl cis-trans isomerase (FK506-binding protein family)	2.2	DAF-16
F42G9.2	*cyn-6*	Cyclophilin type peptidyl-prolyl cis-trans isomerase	1.9	DAF-16
**Protein aggregation and increased sensitivity to hypertonic stress RNAi phenotypes**				
C44H4.2	*let-4*	Extracellular leucine-rich repeat protein; increased protein aggregation [50]	9.9	
ZC373.7	*col-176*	Collagen; increased protein aggregation [51]	9.9	
T23F2.1	*bus-8*	Glycosyltransferase; increased protein aggregation [50]	5.8	
F47F6.1	*lin-42*	PAS domain-containing protein; increased protein aggregation [51]	5.5	SKN-1
C42D8.5	*acn-1*	ACE-like protein; increased protein aggregation [50]	5.4	
ZK783.1	*fbn-1*	Protein homologous to fibrillin; increased sensitivity to hypertonic stress [13]	4.5	DAF-16
H04M03.4	*glf-1*	UDP-galactopyranose mutase; increased protein aggregation; increased sensitivity to hypertonic stress [13]	4.5	DAF-16
W08F4.6	*mlt-8*	Novel protein required for molting; increased protein aggregation [50]	4.4	DAF-16
Y11D7A.9	*Y11D7A*.*9*	Unknown; increased sensitivity to hypertonic stress [13]	4.1	
W05G11.3	*col-88*	Collagen; increased sensitivity to hypertonic stress [13]	3.1	
F58A4.11	*gei-13*	BED finger domain-containing protein; increased protein aggregation [50]	2.8	
C01F1.3	*C01F1*.*3*	Nucleotide-sugar metabolism; increased protein aggregation [50]	2.78	
M03F4.6	*M03F4*.*6*	Unknown; increased protein aggregation [50]	2.8	DAF-16
T19B10.2	*T19B10*.*2*	Unknown; increased protein aggregation; increased sensitivity to hypertonic stress [13]	2.7	DAF-16
W06F12.1	*lit-1*	Serine/threonine kinase; increased protein aggregation [50]	2.2	SKN-1
Y110A2AL.8	*ptc-3*	Patched protein homolog; increased protein aggregation [50]	2.0	
C02F5.7	*C02F5*.*7*	F-box motif-containing protein; resistant to protein aggregation-induced paralysis [49]	2.0	SKN-1
W04H10.3	*nhl-3*	NHL domain-containing protein; increased protein aggregation [52]	1.9	SKN-1
W07E11.1	*W07E11*.*1*	Glutamate metabolism; increased protein aggregation [52]	1.8	
ZK1236.3	*sor-1*	Unknown; increased protein aggregation [50]	1.7	
ZK54.2	*tps-1*	Trehalose 6-phosphate synthase; increased protein aggregation; increased sensitivity to hypertonic stress [52,53]	1.6	DAF-16
M110.5	*dab-1*	Disabled protein homolog; increased protein aggregation [50]	1.5	DAF-16

Twenty-two genes with diverse functions that are not associated with proteostasis in obvious ways were upregulated 1.5- to 9.9-fold in control *osm-9(ok1677)* worms (Table 2). RNAi knockdown of these genes results in increased protein aggregation, reduced resistance to hypertonic stress and/or changes in sensitivity to protein aggregation-induced paralysis.

*osm-9(ok1677)* worms placed under hypertonic stress also exhibited significant (P<0.05) differential upregulation of genes associated with proteostasis (Table 3). Eight of these genes have RNAi phenoptypes of increased protein aggregation, altered sensitivity to protein aggregation and/or decreased hypertonic stress resistance.

**Table 3:** Putative proteostasis genes differentially upregulated in *osm-9(ok1677)* worms following a 6 h exposure to 200 mM NaCl.

Sequence name	Gene	Brief description and RNAi phenotypes	*osm-9(ok1677)* vs. WT fold change	modENCODE ChIP-Seq Peaks
**Protein degradation**				
H19M22.3	*H19M22*.*3*	Metalloproteinase	3.7	
F44B9.1	*dpf-6*	Peptidase	3.5	
F41E6.6	*tag-196*	Cathepsin-like cysteine protease	2.0	DAF-16
LLC1.1	*tra-3*	Calpain type protease; lysosome function	1.8	
D2030.2	*D2030*.*2*	Clp protease	1.5	DAF-16
K11D2.2	*asah-1*	Acid ceramidase; lysosome function	2.3	
F27E5.1	*F27E5*.*1*	Acid ceramidase; lysosome function	1.5	
Y38F2AL.4	*vha-3*	Vacuolar proton ATPase subunit; lysosome function; increased protein aggregation and increased sensitivity to hypertonic stress [13]	1.6	
Y37E11AR.2	*siah-1*	E3 ubiquitin ligase	2.8	SKN-1
**Protein folding**				
Y18D10A.25	*fkb-8*	Peptidyl-prolyl cis-trans isomerase (FK506-binding protein family)	2.0	
**Protein aggregation and increased sensitivity to hypertonic stress RNAi phenotypes**				
ZC373.7	*col-176*	Collagen; increased protein aggregation [51]	5.4	
C44H4.2	*let-4*	Extracellular leucine-rich repeat protein; increased protein aggregation [50]	3.6	
F52B11.3	*noah-2*	PAN and ZP domain containing protein; increased protein aggregation [50]	2.7	
ZK783.1	*fbn-1*	Protein homologous to fibrillin; increased sensitivity to hypertonic stress [13]	2.4	DAF-16
C01F1.3	*C01F1*.*3*	Nucleotide-sugar metabolism; increased protein aggregation [13]	2.2	
C02F5.7	*C02F5*.*7*	F-box motif-containing protein; resistant to protein aggregation-induced paralysis [49]	1.9	SKN-1
W07E11.1	*W07E11*.*1*	Glutamate metabolism; increased protein aggregation [49]	1.6	

The transcription factors DAF-16 and SKN-1 play important roles in regulating stress resistance, longevity and proteostasis [54–57]. Target genes for DAF-16 have been identified by Tepper et al. [33] using chromatin immunoprecipitation sequencing (ChIP-seq) and other functional genomic datasets. The modENCODE project [58] has utilized ChIP-seq to identify SKN-1 regulated genes [34] (http://modencode.org). As shown in Tables 2 and 3, 48% and 29%, respectively, of the genes upregulated in *osm-9(ok1677)* worms have binding sites for these two transcription factors. DAF-16 and SKN-1 targets were enriched significantly (P<0.01) in the upregulated genes identified in *osm-9(ok1677)* worms under control conditions (Table 2). Twenty-six of the 53 (i.e., 49%) upregulated genes are predicted DAF-16 and SKN-1 targets compared to 5,886 predicted target genes in the 17,737 (i.e., 33%) genes expressed in wild type and *osm-9(ok1677)* worms.

## Discussion

ASH chemosensory neurons mediate avoidance behavior to hypertonic solutions as well as other noxious chemical and mechanical stimuli [59]. Animals harboring mutations that disrupt hypertonic avoidance behavior (Fig 1 and Fig 3A) or in which ASH neurons have been ablated (Fig 1) exhibit improved survival during hypertonic stress. *osm-9*, *osm-12* and *sra-6*, which drives ASH ablation [21], are expressed in ASH as well as other neuron types [39]. The only cell types in which expression of these three genes has been shown to overlap are ASH and ASI chemosensory neurons [39]. ASI neurons function in dauer formation, chemotaxis and navigation [59]. However, genetic ablation of ASI or loss-of-function mutation of a gene required for ASI function had no effect on survival on hypertonic environments (Fig 1D). Therefore, we propose that loss of ASH neuron functions required for avoidance of hypertonic environments enhances basal hypertonic stress resistance.

Enhanced hypertonic stress resistance in animals with defective ASH neuron function is not due to alterations in systemic volume regulation or glycerol metabolism (Fig 2). These conclusions are consistent with our earlier findings demonstrating that dramatic reductions in glycerol accumulation do not reduce short term survival during hypertonic stress [17]. As we have shown previously, proteostasis capacity is a critical limiting factor for survival during hypertonic stress [13]. Consistent with this, we found that *osm-9(ok1677)* mutant and *osm-9(RNAi)* worms exhibit reduced protein damage during hypertonic stress (Figs 3 and 4).

Improved proteostasis during hypertonic stress in *osm-9(ok1677)* mutant worms may be due in part to the upregulation of genes that play known or likely roles in protein synthesis, folding and degradation (Tables 2 and 3). RNAi phenotypes of various upregulated genes such as the vacuolar proton ATPase *vha-3*, the E3 ubiquitin-protein ligase *C17H11*.*6* and the eukaryotic translation elongation factor *eef-1A*.*2* include increased protein aggregation, increased sensitivity to hypertonic stress and increased sensitivity to protein aggregation-induced paralysis [13,49,50].

Several studies have shown that diverse proteins with no known role in proteostasis slow or prevent protein aggregation [60–64]. Many genes upregulated in *osm-9(ok1677)* worms also have no known proteostasis function, but protein aggregation is increased by RNAi knockdown of their activity. Two of these genes showed particularly striking upregulation. Expression of *col-176* and *let-4* was increased compared to wild type animals 9.9- and 3.6-fold and 9.9- and 5.4-fold under control and hypertonic stress conditions, respectively (Table 2 and 3).

*col-176* encodes a cuticle collagen. RNAi silencing of this gene increases aggregation of misfolding-prone mutant human SOD1 protein [51]. Several in vitro studies have shown that collagen interacts with aggregation prone proteins and slows the growth of toxic amyloid fibrils [60–62]. Increased expression of *col-176* could minimize hypertonic stress induced protein damage by similar mechanisms. In addition, various collagens have been shown previously to play an important role in osmotic stress signaling in *C*. *elegans* [17,37]. Remodeling of the extracellular matrix by increased expression of *col-176* may alter signaling processes that directly or indirectly regulate proteostasis mechanisms activated in response to hypertonic stress.

*let-4* encodes a leucine-rich repeat protein expressed on the apical surface of epidermal and epithelial cells [65]. Knockdown of *let-4* increases Q35::YFP aggregation [50]. RNAi screening in mammalian neuronal cells identified a leucine-rich repeat protein that regulates mutant huntingtin protein aggregation [66]. The leucine-rich repeat is a protein motif that mediates protein-protein interactions and thus plays important roles in diverse cellular processes [67]. Proteins containing leucine-rich repeat motifs function in protein degradation [68,69] and have been implicated in protein aggregation and impaired autophagy associated with Parkinson’s Disease [70].

In *C*. *elegans*, *let-4* likely plays an important role in maintaining epithelial integrity [65]. Disruption of epithelial barrier function is expected to disrupt salt and water homeostasis, which in turn could lead to increased protein aggregation. Increased *let-4* expression in *osm-9(ok1677)* worms may improve epithelial barrier function under hypertonic conditions. While speculative, it is also possible that leucine-rich repeat proteins like collagens may preferentially bind with denatured proteins and slow aggregate formation.

It should be stressed here that while RNAseq has been shown to be an accurate tool for assessing differential gene expression [71–73], additional studies will be needed confirm the results shown in Tables 2 and 3. Furthermore, it is conceivable that the changes in gene expression and reduced protein damage observed in *osm-9(ok1677)* mutant worms may be unique to this strain and not to loss of *osm-9* function per se. Therefore, it will also be important to determine if other worm strains with defective ASH neuron function show similar differences in gene expression and proteostasis capacity. If our findings are confirmed, it will be necessary to determine the cellular locations of altered gene expression and the mechanisms by which differentially expressed genes confer increased hypertonic stress resistance.

An animal’s nervous system plays a critical role in detecting its complex and constantly changing external environment and integrating those environmental cues to coordinately regulate multiple physiological processes. Because of its relatively simple nervous system and genetic and molecular tractability, *C*. *elegans* has begun to provide insights into the underlying molecular mechanisms. For example, Prahlad and Morimoto [9] have shown that calcium activated dense core vesicle neurosecretion from *C*. *elegans* thermosensory neurons normally suppresses heat shock transcription factor 1 (HSF-1) dependent chaperone expression in muscle and intestinal cells. Signaling from ASH as well as ASI sensory neurons negatively regulates the innate immune response by MAP kinase dependent inhibition of the expression of genes that are part of the noncanonical unfolded protein response pathway [74]. In a similar fashion, osmosensory signaling from ASH neurons could regulate osmoprotective gene expression in non-neuronal cells. However, additional studies will be needed to more thoroughly test this possibility.

In summary, we have demonstrated that defects in ASH mediated hypertonic avoidance behavior are associated with enhanced hypertonic stress resistance and improved proteostasis. Our findings are consistent with a growing body of work demonstrating that intercellular communication between neuronal and non-neuronal cells plays a critical role in integrating cellular stress responses with other organismal physiological demands and associated energy costs.

## Supporting Information

S1 Appendix Raw survival data. (XLSX)

## References

[1] Balch WE, Morimoto RI, Dillin A, Kelly JW. Adapting proteostasis for disease intervention. Science 2008 319:916–9. 10.1126/science.1141448 18276881

[2] Cohen E, Dillin A. The insulin paradox: aging, proteotoxicity and neurodegeneration. Nat Rev Neurosci 2008 9:759–67. 10.1038/nrn2474 18769445 PMC2692886

[3] Powers ET, Morimoto RI, Dillin A, Kelly JW, Balch WE. Biological and chemical approaches to diseases of proteostasis deficiency. Annu Rev Biochem 2009 78:959–91. 10.1146/annurev.biochem.052308.114844 19298183

[4] Ghosh K, Dill K. Cellular proteomes have broad distributions of protein stability. Biophys J 2010 99:3996–4002. 10.1016/j.bpj.2010.10.036 21156142 PMC3000515

[5] Goldschmidt L, Teng PK, Riek R, Eisenberg D. Identifying the amylome, proteins capable of forming amyloid-like fibrils. Proc Natl Acad Sci U S A 2010 107:3487–92. 10.1073/pnas.0915166107 20133726 PMC2840437

[6] Koga H, Kaushik S, Cuervo AM. Protein homeostasis and aging: The importance of exquisite quality control. Ageing Res Rev 2011 10:205–15. 10.1016/j.arr.2010.02.001 20152936 PMC2888802

[7] Labbadia J, Morimoto RI. Proteostasis and longevity: when does aging really begin? F1000Prime Rep 2014 6:7 eCollection;%2014.:7. 10.12703/P6-7 24592319 PMC3914504

[8] Prahlad V, Cornelius T, Morimoto RI. Regulation of the cellular heat shock response in *Caenorhabditis elegans* by thermosensory neurons. Science 2008 320:811–4. 10.1126/science.1156093 18467592 PMC3429343

[9] Prahlad V, Morimoto RI. Neuronal circuitry regulates the response of *Caenorhabditis elegans* to misfolded proteins. Proc Natl Acad Sci U S A 2011 108:14204–9. 10.1073/pnas.1106557108 21844355 PMC3161566

[10] Taylor RC, Dillin A. XBP-1 is a cell-nonautonomous regulator of stress resistance and longevity. Cell 2013 153:1435–47. 10.1016/j.cell.2013.05.042 23791175 PMC4771415

[11] Melo JA, Ruvkun G. Inactivation of conserved *C*. *elegans* genes engages pathogen- and xenobiotic-associated defenses. Cell 2012 149:452–66. 10.1016/j.cell.2012.02.050 22500807 PMC3613046

[12] Lamitina ST, Morrison R, Moeckel GW, Strange K. Adaptation of the nematode *Caenorhabditis elegans* to extreme osmotic stress. Am J Physiol Cell Physiol 2004 286:C785–C791. 14644776 10.1152/ajpcell.00381.2003

[13] Choe KP, Strange K. Genome-wide RNAi screen and in vivo protein aggregation reporters identify degradation of damaged proteins as an essential hypertonic stress response. Am J Physiol Cell Physiol 2008 295:C1488–C1498. 10.1152/ajpcell.00450.2008 18829898 PMC2603564

[14] Burkewitz K, Choe K, Strange K. Hypertonic stress induces rapid and widespread protein damage in *C*. *elegans*. Am J Physiol Cell Physiol 2011 301:C566–C576. 10.1152/ajpcell.00030.2011 21613604 PMC3174568

[15] Burkewitz K, Choe KP, Choung-Hee LE, Deonarine A, Strange K. Characterization of the proteostasis roles of glycerol accumulation, protein degradation and protein synthesis during osmotic stress in *C*. *elegans*. PLoS One 2012 7:e34153 10.1371/journal.pone.0034153 22470531 PMC3314593

[16] Kim H, Strange K. Changes in translation rate modulate stress-induced damage of diverse proteins. Am J Physiol Cell Physiol 2013 305:C1257–C1264. 10.1152/ajpcell.00176.2013 24153430 PMC3882363

[17] Lamitina T, Huang CG, Strange K. Genome-wide RNAi screening identifies protein damage as a regulator of osmoprotective gene expression. Proc Natl Acad Sci U S A 2006 103:12173–8. 16880390 10.1073/pnas.0602987103 PMC1567714

[18] Lee EC, Strange K. GCN-2 dependent inhibition of protein synthesis activates osmosensitive gene transcription via WNK and Ste20 kinase signaling. Am J Physiol Cell Physiol 2012 303:C1269–C1277. 10.1152/ajpcell.00294.2012 23076791 PMC3532491

[19] Liedtke W, Tobin DM, Bargmann CI, Friedman JM. Mammalian TRPV4 (VR-OAC) directs behavioral responses to osmotic and mechanical stimuli in *Caenorhabditis elegans*. Proc Natl Acad Sci U S A 2003 100:14531–6. 14581619 10.1073/pnas.2235619100 PMC304114

[20] Colbert HA, Smith TL, Bargmann CI. OSM-9, a novel protein with structural similarity to channels, is required for olfaction, mechanosensation, and olfactory adaptation in *Caenorhabditis elegans*. J Neurosci 1997 17:8259–69. 9334401 10.1523/JNEUROSCI.17-21-08259.1997 PMC6573730

[21] Yoshida K, Hirotsu T, Tagawa T, Oda S, Wakabayashi T, Iino Y, et al Odour concentration-dependent olfactory preference change in *C*. *elegans*. Nat Commun 2012 3:739 10.1038/ncomms1750 22415830

[22] Calahorro F, Alejandre E, Ruiz-Rubio M. Osmotic avoidance in *Caenorhabditis elegans*: synaptic function of two genes, orthologues of human NRXN1 and NLGN1, as candidates for autism. J Vis Exp 2009 11:1616. 10.3791/1616 PMC3346054 20010541

[23] Brenner S. The genetics of *Caenorhabditis elegans*. Genetics 1974 77:71–94. 4366476 10.1093/genetics/77.1.71 PMC1213120

[24] Anderson LL, Mao X, Scott BA, Crowder CM. Survival from hypoxia in *C*. *elegans* by inactivation of aminoacyl-tRNA synthetases. Science 2009 323:630–3. 10.1126/science.1166175 19179530 PMC3739282

[25] The Nematode *Caenorhabditis elegans* Cold Spring Harbor: Cold Spring Harbor Laboratory Press; 1988.

[26] Choi S, Chatzigeorgiou M, Taylor KP, Schafer WR, Kaplan JM. Analysis of NPR-1 reveals a circuit mechanism for behavioral quiescence in *C*. *elegans*. Neuron 2013 78:869–80. 10.1016/j.neuron.2013.04.002 23764289 PMC3683153

[27] Trapnell C, Pachter L, Salzberg SL. TopHat: discovering splice junctions with RNA-Seq. Bioinformatics 2009 25:1105–11. 10.1093/bioinformatics/btp120 19289445 PMC2672628

[28] Flicek P, Amode MR, Barrell D, Beal K, Billis K, Brent S, et al Ensembl 2014. Nucleic Acids Res 2014 42:D749–D755. 10.1093/nar/gkt1196 24316576 PMC3964975

[29] Trapnell C, Williams BA, Pertea G, Mortazavi A, Kwan G, van Baren MJ, et al Transcript assembly and quantification by RNA-Seq reveals unannotated transcripts and isoform switching during cell differentiation. Nat Biotechnol 2010 28:511–5. 10.1038/nbt.1621 20436464 PMC3146043

[30] Robinson MD, McCarthy DJ, Smyth GK. edgeR: a Bioconductor package for differential expression analysis of digital gene expression data. Bioinformatics 2010 26:139–40. 10.1093/bioinformatics/btp616 19910308 PMC2796818

[31] Harris TW, Baran J, Bieri T, Cabunoc A, Chan J, Chen WJ, et al WormBase 2014: new views of curated biology. Nucleic Acids Res 2014 42:D789–D793. 10.1093/nar/gkt1063 24194605 PMC3965043

[32] Kinsella RJ, Kahari A, Haider S, Zamora J, Proctor G, Spudich G, et al Ensembl BioMarts: a hub for data retrieval across taxonomic space. Database (Oxford) 2011 2011:bar030. 21785142 10.1093/database/bar030 PMC3170168

[33] Tepper RG, Ashraf J, Kaletsky R, Kleemann G, Murphy CT, Bussemaker HJ. PQM-1 complements DAF-16 as a key transcriptional regulator of DAF-2-mediated development and longevity. Cell 2013 154:676–90. 10.1016/j.cell.2013.07.006 23911329 PMC3763726

[34] Niu W, Lu ZJ, Zhong M, Sarov M, Murray JI, Brdlik CM, et al Diverse transcription factor binding features revealed by genome-wide ChIP-seq in *C*. *elegans*. Genome Res 2011 21:245–54. 10.1101/gr.114587.110 21177963 PMC3032928

[35] Wheeler JM, Thomas JH. Identification of a novel gene family involved in osmotic stress response in *Caenorhabditis elegans*. Genetics 2006 174:1327–36. 16980399 10.1534/genetics.106.059089 PMC1667073

[36] Rohlfing AK, Miteva Y, Hannenhalli S, Lamitina T. Genetic and physiological activation of osmosensitive gene expression mimics transcriptional signatures of pathogen infection in *C*. *elegans*. PLoS One 2010 5:e9010 10.1371/journal.pone.0009010 20126308 PMC2814864

[37] Rohlfing AK, Miteva Y, Moronetti L, He L, Lamitina T. The *Caenorhabditis elegans* mucin-like protein OSM-8 negatively regulates osmosensitive physiology via the transmembrane protein PTR-23. PLoS Genet 2011 7:e1001267 10.1371/journal.pgen.1001267 21253570 PMC3017116

[38] Blacque OE, Reardon MJ, Li C, McCarthy J, Mahjoub MR, Ansley SJ, et al Loss of *C*. *elegans* BBS-7 and BBS-8 protein function results in cilia defects and compromised intraflagellar transport. Genes Dev 2004 18:1630–42. 15231740 10.1101/gad.1194004 PMC443524

[39] WormBase web site, Version WS248. 2015.

[40] Jansen G, Thijssen KL, Werner P, van der Horst M, Hazendonk E, Plasterk RH. The complete family of genes encoding G proteins of *Caenorhabditis elegans*. Nat Genet 1999 21:414–9. 10192394 10.1038/7753

[41] Beverly M, Anbil S, Sengupta P. Degeneracy and neuromodulation among thermosensory neurons contribute to robust thermosensory behaviors in *Caenorhabditis elegans*. J Neurosci 2011 31:11718–27. 10.1523/JNEUROSCI.1098-11.2011 21832201 PMC3167209

[42] Morley JF, Brignull HR, Weyers JJ, Morimoto RI. The threshold for polyglutamine-expansion protein aggregation and cellular toxicity is dynamic and influenced by aging in *Caenorhabditis elegans*. Proc Natl Acad Sci U S A 2002 99:10417–22. 12122205 10.1073/pnas.152161099 PMC124929

[43] Brown CR, Hong-Brown LQ, Welch WJ. Correcting temperature-sensitive protein folding defects. J Clin Invest 1997 99:1432–44. 9077553 10.1172/JCI119302 PMC507959

[44] Gidalevitz T, Ben Zvi A, Ho KH, Brignull HR, Morimoto RI. Progressive disruption of cellular protein folding in models of polyglutamine diseases. Science 2006 311:1471–4. 16469881 10.1126/science.1124514

[45] Van Dyk TK, Gatenby AA, LaRossa RA. Demonstration by genetic suppression of interaction of GroE products with many proteins. Nature 1989 342:451–3. 2573840 10.1038/342451a0

[46] Calixto A, Chelur D, Topalidou I, Chen X, Chalfie M. Enhanced neuronal RNAi in *C*. *elegans* using SID-1. Nat Methods 2010 7:554–9. 10.1038/nmeth.1463 20512143 PMC2894993

[47] Chandler-Brown D, Choi H, Park S, Ocampo BR, Chen S, Le A, et al Sorbitol treatment extends lifespan and induces the osmotic stress response in *Caenorhabditis elegans*. Front Genet 2015 10 27;6:316 10.3389/fgene.2015.00316 eCollection;%2015.:316. 26579191 PMC4621483

[48] Wang X, Thomas B, Sachdeva R, Arterburn L, Frye L, Hatcher PG, et al Mechanism of arylating quinone toxicity involving Michael adduct formation and induction of endoplasmic reticulum stress. Proc Natl Acad Sci U S A 2006 103:3604–9. 16505371 10.1073/pnas.0510962103 PMC1450130

[49] Mehta R, Steinkraus KA, Sutphin GL, Ramos FJ, Shamieh LS, Huh A, et al Proteasomal regulation of the hypoxic response modulates aging in *C*. *elegans*. Science 2009 324:1196–8. 10.1126/science.1173507 19372390 PMC2737476

[50] Nollen EA, Garcia SM, van Haaften G, Kim S, Chavez A, Morimoto RI, et al Genome-wide RNA interference screen identifies previously undescribed regulators of polyglutamine aggregation. Proc Natl Acad Sci U S A 2004 101:6403–8. 15084750 10.1073/pnas.0307697101 PMC404057

[51] Wang J, Farr GW, Hall DH, Li F, Furtak K, Dreier L, et al An ALS-linked mutant SOD1 produces a locomotor defect associated with aggregation and synaptic dysfunction when expressed in neurons of *Caenorhabditis elegan*s. PLoS Genet 2009 5:e1000350 10.1371/journal.pgen.1000350 19165329 PMC2621352

[52] Hamamichi S, Rivas RN, Knight AL, Cao S, Caldwell KA, Caldwell GA. Hypothesis-based RNAi screening identifies neuroprotective genes in a Parkinson's disease model. Proc Natl Acad Sci U S A 2008 105:728–33. 10.1073/pnas.0711018105 18182484 PMC2206604

[53] Lamitina ST, Strange K. Transcriptional targets of the DAF-16 insulin signaling pathwayprotect *C*. *elegans* from extreme hypertonic stress. Am J Physiol Cell Physiol 2004 288:467–74. 10.1152/ajpcell.00451.2004 15496475

[54] Oh KH, Kim H. Reduced IGF signaling prevents muscle cell death in a *Caenorhabditis elegans* model of muscular dystrophy. Proc Natl Acad Sci U S A 2013 110:19024–9. 10.1073/pnas.1308866110 24191049 PMC3839758

[55] Matilainen O, Arpalahti L, Rantanen V, Hautaniemi S, Holmberg CI. Insulin/IGF-1 signaling regulates proteasome activity through the deubiquitinating enzyme UBH-4. Cell Rep 2013 3:1980–95. 10.1016/j.celrep.2013.05.012 23770237

[56] Wolff S, Dillin A. The trifecta of aging in *Caenorhabditis elegans*. Exp Gerontol 2006 41:894–903. 16919905 10.1016/j.exger.2006.06.054

[57] Sykiotis GP, Bohmann D. Stress-activated cap'n'collar transcription factors in aging and human disease. Sci Signal 2010 3:re3 10.1126/scisignal.3112re3 20215646 PMC2991085

[58] Celniker SE, Dillon LA, Gerstein MB, Gunsalus KC, Henikoff S, Karpen GH, et al Unlocking the secrets of the genome. Nature 2009 459:927–30. 10.1038/459927a 19536255 PMC2843545

[59] Bargmann CI. Chemosensation in C. elegans. WormBook 2006 10 25;1–29. 10.1895/wormbook.1.123.1 PMC4781564 18050433

[60] Dubey K, Kar K. Type I collagen prevents amyloid aggregation of hen egg white lysozyme. Biochem Biophys Res Commun 2014 448:480–4. 10.1016/j.bbrc.2014.04.135 24802405

[61] Parmar AS, Nunes AM, Baum J, Brodsky B. A peptide study of the relationship between the collagen triple-helix and amyloid. Biopolymers 2012 97:795–806. 10.1002/bip.22070 22806499 PMC3400121

[62] Kakuyama H, Soderberg L, Horigome K, Winblad B, Dahlqvist C, Naslund J, et al CLAC binds to aggregated Aβ and Aβ fragments, and attenuates fibril elongation. Biochemistry 2005 44:15602–9. 16300410 10.1021/bi051263e

[63] Luo J, Warmlander SK, Graslund A, Abrahams JP. Non-chaperone proteins can inhibit aggregation and cytotoxicity of Alzheimer amyloid β peptide. J Biol Chem 2014 289:27766–75. 10.1074/jbc.M114.574947 25100721 PMC4183812

[64] Stanyon HF, Viles JH. Human serum albumin can regulate amyloid-β peptide fiber growth in the brain interstitium: implications for Alzheimer disease. J Biol Chem 2012 287:28163–8. 10.1074/jbc.C112.360800 22718756 PMC3431649

[65] Mancuso VP, Parry JM, Storer L, Poggioli C, Nguyen KC, Hall DH, et al Extracellular leucine-rich repeat proteins are required to organize the apical extracellular matrix and maintain epithelial junction integrity in *C*. *elegans*. Development 2012 139:979–90. 10.1242/dev.075135 22278925 PMC3274359

[66] Yamanaka T, Wong HK, Tosaki A, Bauer PO, Wada K, Kurosawa M, et al Large-scale RNA interference screening in mammalian cells identifies novel regulators of mutant huntingtin aggregation. PLoS One 2014 9:e93891 10.1371/journal.pone.0093891 24705917 PMC3976342

[67] Kobe B, Kajava AV. The leucine-rich repeat as a protein recognition motif. Curr Opin Struct Biol 2001 11:725–32. 11751054 10.1016/s0959-440x(01)00266-4

[68] Muniz JR, Guo K, Kershaw NJ, Ayinampudi V, von DF, Babon JJ, et al Molecular architecture of the ankyrin SOCS box family of Cul5-dependent E3 ubiquitin ligases. J Mol Biol 2013 425:3166–77. 10.1016/j.jmb.2013.06.015 23806657 PMC3779351

[69] Spaich S, Will RD, Just S, Spaich S, Kuhn C, Frank D, et al F-box and leucine-rich repeat protein 22 is a cardiac-enriched F-box protein that regulates sarcomeric protein turnover and is essential for maintenance of contractile function in vivo. Circ Res 2012 111:1504–16. 10.1161/CIRCRESAHA.112.271007 22972877

[70] Dzamko N, Zhou J, Huang Y, Halliday GM. Parkinson's disease-implicated kinases in the brain; insights into disease pathogenesis. Front Mol Neurosci 20147:57. 10.3389/fnmol.2014.00057 PMC4068290 25009465

[71] A comprehensive assessment of RNA-seq accuracy, reproducibility and information content by the Sequencing Quality Control Consortium. Nat Biotechnol 2014 32:903–14. 10.1038/nbt.2957 25150838 PMC4321899

[72] Jiang L, Schlesinger F, Davis CA, Zhang Y, Li R, Salit M, et al Synthetic spike-in standards for RNA-seq experiments. Genome Res 2011 21:1543–51. 10.1101/gr.121095.111 21816910 PMC3166838

[73] Rapaport F, Khanin R, Liang Y, Pirun M, Krek A, Zumbo P, et al Comprehensive evaluation of differential gene expression analysis methods for RNA-seq data. Genome Biol 2013 14:R95 24020486 10.1186/gb-2013-14-9-r95 PMC4054597

[74] Sun J, Singh V, Kajino-Sakamoto R, Aballay A. Neuronal GPCR controls innate immunity by regulating noncanonical unfolded protein response genes. Science 2011 332:729–32. 10.1126/science.1203411 21474712 PMC3125668

